# A Diagnosis of Normocalcemic Primary Hyperparathyroidism Prompted by “Salt and Pepper” Lesions of the Calvarium

**DOI:** 10.1016/j.aace.2021.07.004

**Published:** 2021-08-03

**Authors:** Simrun K. Bal, Meredith J. Sorensen, Andrew Robert Crawford

**Affiliations:** 1 Department of Medicine, Dartmouth-Hitchcock Medical Center, Lebanon, New Hampshire; 2 Section of Endocrine Surgery, Department of Surgery, Dartmouth-Hitchcock Medical Center, Lebanon, New Hampshire; 3 Section of Endocrinology, Department of Medicine, Dartmouth-Hitchcock Medical Center, Lebanon, New Hampshire

**Keywords:** normocalcemic primary hyperparathyroidism, parathyroid hormone, salt and pepper lesions, PTH, parathyroid hormone

## Abstract

**Background:**

We report a case of normocalcemic primary hyperparathyroidism, a diagnosis prompted by radiographic “salt and pepper” calvarial lesions, typically described in hypercalcemic primary hyperparathyroidism or secondary hyperparathyroidism.

**Case Report:**

A 60-year-old woman noticed indentations of her scalp and presented to her primary care provider. Radiography of the calvarium demonstrated granular “salt and pepper” lesions, prompting investigation. The patient was found to have an elevated parathyroid hormone (PTH) level of 79 pg/mL (reference range, 14-54 pg/mL) and a normal albumin-corrected calcium level of 9.8 mg/dL (reference range, 8.6-10.4 mg/dL). She was referred to our endocrine clinic and described having bone aches, fevers, leg cramps, and a remote history of nephrolithiasis. Her physical examination revealed hypertension. Repeat laboratory evaluation confirmed elevated PTH and normal albumin-corrected calcium. Secondary causes of hyperparathyroidism were ruled out. Her 25-hydroxyvitamin D level was 35 ng/mL (reference range, 30-100 ng/mL), with a normal creatinine level (0.73 mg/dL; reference range, 0.5-0.99 mg/dL). The patient underwent ultrasound and sestamibi scintigraphy, with uptake in the right inferior thyroid pole. She was found to have a 6-mm parathyroid adenoma and underwent a targeted parathyroidectomy, with normalization of serum PTH.

**Discussion:**

Many cases of normocalcemic primary hyperparathyroidism are diagnosed in asymptomatic patients presenting with low bone mass; however, imaging prompted this patient's evaluation. Ultimately, the calvarial lesions were thought secondary to bone resorption from increased osteoclast activity.

**Conclusion:**

This case highlights an atypical presentation of normocalcemic primary hyperparathyroidism in that the evaluation was precipitated by unexpected radiographic evidence of metabolic bone disease, rather than by symptoms or biochemical studies.

## Introduction

Primary hyperparathyroidism is a disorder characterized by the inappropriate secretion of parathyroid hormone (PTH), leading to skeletal or renal disease in the context of altered calcium homeostasis, typically with hypercalcemia.^1^ However, with the advent of sensitive diagnostic assays, patients are increasingly being identified as having primary hyperparathyroidism with consistently normal serum calcium concentrations in the absence of secondary causes of hyperparathyroidism. In most cases of normocalcemic primary hyperparathyroidism, patients are asymptomatic or have been referred for further evaluation after being found to have low bone mass.^2^ This case report, however, describes a unique case of a patient diagnosed with normocalcemic primary hyperparathyroidism after being found to have granular “salt and pepper” lesions of the calvarium on radiography.

Our objectives were as follows: (1) describe the typical presentation of patients with normocalcemic primary hyperparathyroidism, (2) review the relevant pathophysiology surrounding bone disease in primary hyperparathyroidism, and (3) describe differences in clinical characteristics between normocalcemic and hypercalcemic patients with primary hyperparathyroidism.

## Case Report

**Fig. 1:**
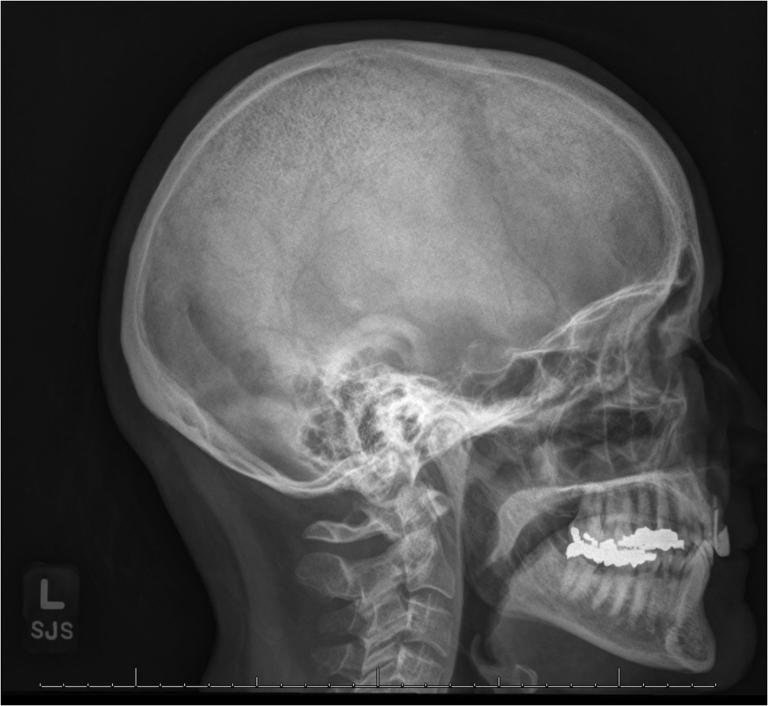
Radiograph of the lateral aspect of the calvarium. The radiograph of the lateral aspect of the calvarium demonstrates characteristic “salt and pepper” lesions with a granular appearance.

A 64-year-old postmenopausal woman presented to her primary care provider after she self-palpated soft depressions in her scalp. Her medical history was notable for osteoarthritis, diverticulitis, hyperlipidemia, and impaired fasting glucose. Her physical examination in the primary care setting was notable for 3 soft, small indentations in the superior skull. A plain film radiograph of the calvarium was notable for a granular appearance with “salt and pepper” lesions (Fig. 1). A preliminary biochemical evaluation was significant for elevated PTH level (79 pg/mL; normal range, 14-54 pg/mL). Serum calcium level was 9.6 mg/dL (normal range, 8.6-10.4 mg/dL) and corrected to 9.8 mg/dL with an albumin level of 3.8 g/dL (normal range, 3.6-5.1 g/dL). Serum creatinine level was also normal (0.75 mg/dL; normal range, 0.5-0.99 mg/dL). With these findings, the patient was referred to the endocrine clinic at our institution.

During her evaluation in our clinic, upon further questioning, the patient gave a history of bone aches, occasional fevers, nausea, mild cognitive decline, and leg cramps and a remote history of nephrolithiasis (12 years prior to presentation). A recent renal ultrasound scan was negative for nephrolithiasis. She had no family history of endocrinopathies, including no calcium disorders, parathyroid disease, pituitary disease, or pancreatic disease. She denied the use of biotin or calcium-containing supplementations.

**Fig. 2:**
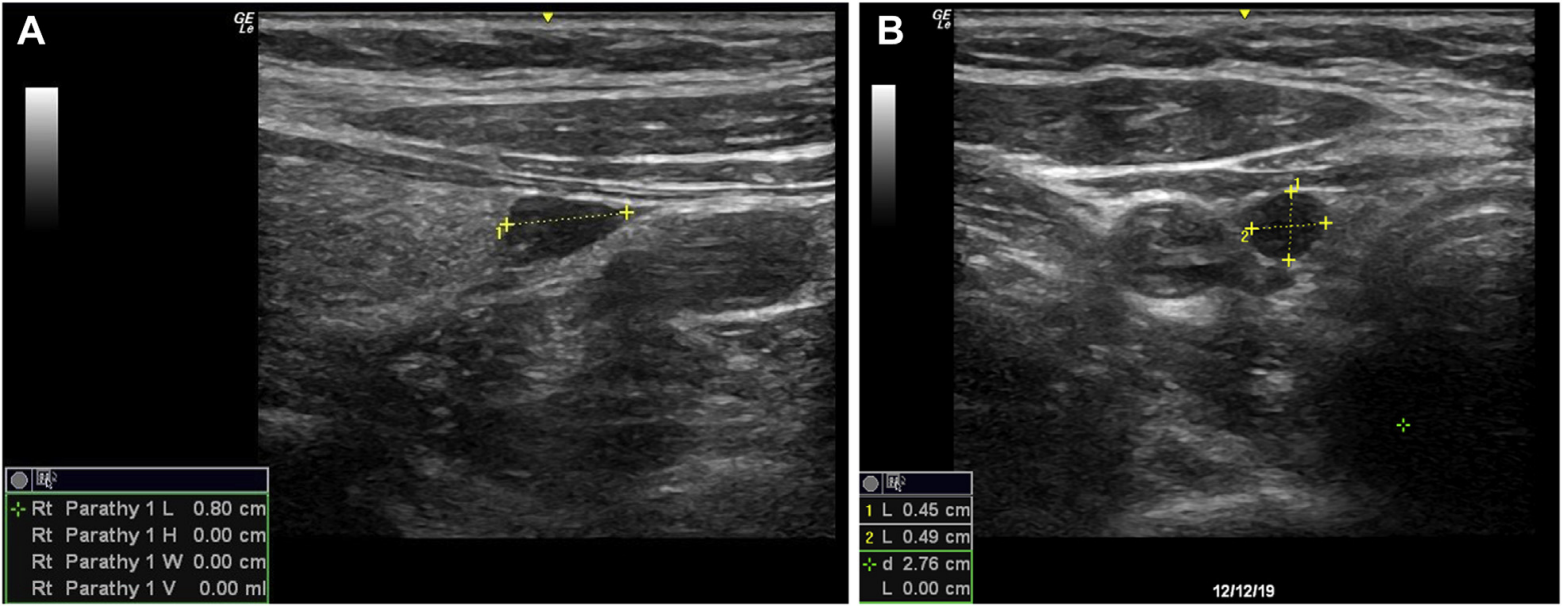
Ultrasound imaging of the parathyroid adenoma. The patient’s ultrasound scan revealed a 6-mm adenoma of the lower portion of the right parathyroid gland. *A*, Sagittal orientation. *B*, Transverse orientation.

**Fig. 3:**
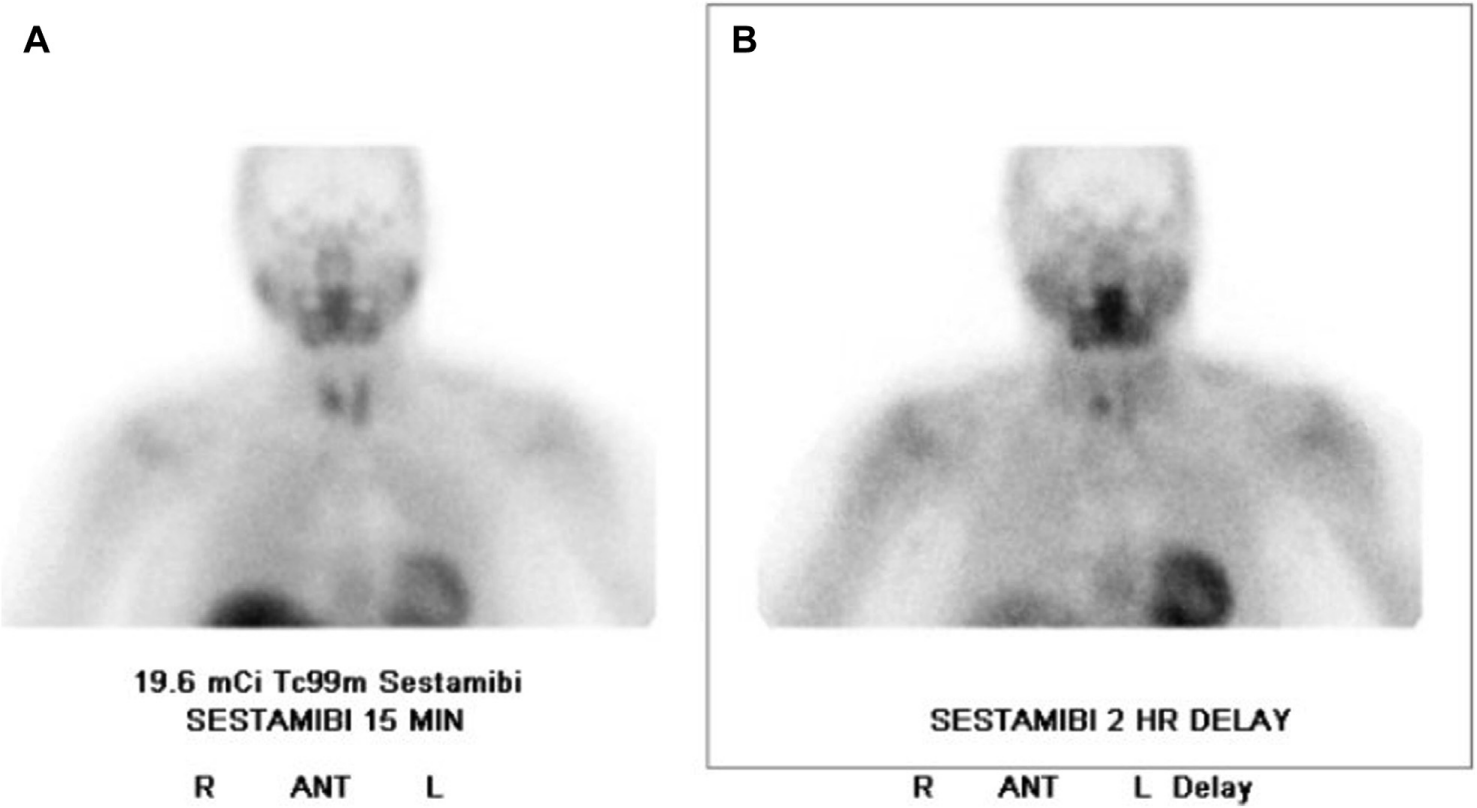
Patient’s sestamibi scintigraphy. Scintigraphy of the parathyroid glands demonstrates the accumulation of the isotope in the lower portion of the right parathyroid. *A,* Early scan at 15 minutes. *B,* Delayed scan at 2 hours. *ANT* = anterior view; *L* = left; *R* = right.

Physical examination was notable for uncontrolled hypertension, with a peak blood pressure of 211/60 mm Hg. A skeletal survey demonstrated a consistent granular appearance of the skull, without focal lytic lesions and with mild demineralization. Biochemical evaluation was again notable for an elevated PTH level (71 pg/mL), normal albumin-corrected calcium level (9.5 mg/dL), normal phosphorus level (3.7 mg/dL; reference range, 2.5-4.5 mg/dL), normal 25-hydroxyvitamin D level (35 ng/mL), and normal renal function (creatinine level, 0.73 mg/dL). Alkaline phosphatase level was normal (80 units/L; reference range, 33-130 units/L). The 24-hour urine calcium level was elevated (311 mg/24 h; reference range, 35-250 mg/24 h), with a urine calcium-to-creatinine ratio of 262 mg/g (reference range, 30-275 mg/g). Serum protein electrophoresis finding was normal. Bone densitometry demonstrated osteopenia with a T-score of −1.4 at the lumbar spine (reference value, ≥−1; Table). Ultrasound of the neck and sestamibi scintigraphy demonstrated a 6-mm parathyroid adenoma (Fig. 2 and 3). The patient successfully underwent a targeted parathyroidectomy with the removal of an enlarged parathyroid gland weighing 0.26 g. Peak intraoperative PTH level was 361 pg/mL and, after excision and removal, nadir PTH level was 76 pg/mL, measured at 10 and 15 minutes after removal. The postoperative PTH level was 29 pg/mL. One month after parathyroidectomy, the patient reported improvement in bone aches, nausea, and leg cramps. Calcium (9.7 mg/dL) and PTH (47 pg/mL) levels remained normal.

**Table:** Relevant Laboratory Findings That Led to the Diagnosis of Normocalcemic Hyperparathyroidism

Tested parameter	Value	Normal range
Parathyroid hormone, pg/mL	71	14-54
Serum calcium, mg/dL	9.7	8.6-10.4
Albumin-corrected calcium, mg/dL	9.5	8.6-10.4
Albumin, mg/dL	4.5	3.5-5.3
Creatinine, mg/dL	0.73	0.5-0.99
GFR	61	≥60
25-Hydroxyvitamin D	35	30-100
Sodium, mmol/L	142	135-146
Potassium, mmol/L	4.3	3.5-5.3
Phosphorus, mg/dL	3.7	2.5-4.5
Alkaline phosphatase, U/L	80	33-130
Urinary calcium excretion, mg/24 h	311	35-250
Urine calcium:Cr ratio, mg/g	262	30-275
Distal radius BMD, g/cm^2^	0.659	n/a
Distal radius T-score	−0.6	≥−1
Spine L1-L4 BMD, g/cm^2^	0.869	n/a
Lumbar spine T-score	−1.4	≥−1
Femoral neck BMD, g/cm^2^	0.712	n/a
Femoral neck T-score	−1.2	≥−1
Total hip BMD, g/cm^2^	0.83	n/a
Total hip T-score	−0.8	≥−1

Abbreviations: BMD = bone mineral density; Cr = creatinine; GFR = glomerular filtration rate; n/a = not applicable.

## Discussion

Primary hyperparathyroidism is traditionally defined as the inappropriate secretion of PTH leading to a characteristic biochemical profile with overt hypercalcemia, and it is commonly complicated by skeletal and renal manifestations.^1^ In recent years, however, the evolution in biochemical screening tools has enabled a shift in the clinical presentation of primary hyperparathyroidism. The assessment of PTH has progressed over the past 50 years, from the utilization of urinary cyclic adenosine monophosphate (serving as a surrogate for PTH) to the contemporary use of sensitive immunometric assays based on distinct monoclonal antibodies that bind to PTH.^3^^,^^4^ These laboratory-based advances have allowed for the earlier identification of primary hyperparathyroidism in individuals who are normocalcemic and often asymptomatic. Here, we reported a diagnosis of primary hyperparathyroidism in a normocalcemic patient that was prompted initially by the radiographic finding of granular decalcification of the calvarium, suggestive of a “salt and pepper” appearance.

A review of the literature demonstrates that most cases of normocalcemic primary hyperparathyroidism are actually discovered in asymptomatic patients who are referred for evaluation of osteopenia or osteoporosis.^5^ In a 5-year study of 32 patients with normocalcemic hyperparathyroidism, 17 patients were diagnosed with hyperparathyroidism after routine bone densitometry demonstrated osteopenia or osteoporosis.^5^ Three patients were evaluated after having an incidental parathyroid abnormality discovered on neck ultrasound, and 3 patients had nephrolithiasis, prompting further evaluation. No patients were identified on the basis of calvarial lesions.

Another study evaluated 37 individuals with normocalcemic primary hyperparathyroidism, without secondary causes of hyperparathyroidism, with normal renal function and normal 25-hydroxyvitamin D levels and without medications that could contribute to changes in calcium or PTH levels.^6^ The majority were referred after an evaluation for low bone mass revealed an inappropriately elevated PTH level. Upon diagnosis of normocalcemic primary hyperparathyroidism, 57% of patients were found to have osteoporosis, 11% had fragility fractures, and 14% had nephrolithiasis.^6^ This study demonstrated that normocalcemia is not associated with an indolent disease state; in fact, patients can have distinct clinical manifestations.

Although the classic clinical features of hyperparathyroidism can include nephrolithiasis or fracture, there are also subtle manifestations of hyperparathyroidism, including hypertension, a major cardiovascular risk factor demonstrated in our patient.^7^ Hypertension has commonly been reported in the literature surrounding hyperparathyroidism.^7^^,^^8^ In a study of more than 1000 patients with primary hyperparathyroidism, parathyroidectomy (with associated correction of hyperparathyroidism) was associated with a significant decrease in both systolic and diastolic blood pressures among hypertensive patients.^7^ Indeed, a recent study featuring more than 2000 patients with hyperparathyroidism demonstrated that parathyroidectomy was associated with improvements in mean arterial pressure and a reduction in the need for antihypertensive medications among postparathyroidectomy patients.^9^

In our patient, radiographic findings of the calvarium precipitated the diagnostic evaluation of hyperparathyroidism. Typically, hypersecretion of PTH is associated with increased osteoclastic activity, leading to fibrovascular replacement of the marrow and demineralization of bone.^1^ This tends to present as bone loss at sites of cortical bone (such as the distal radius) rather than at those of trabecular bone.^10^ On comparing normocalcemic subjects with primary hyperparathyroidism to those with hypercalcemia, both groups exhibited catabolic activity at the cortical and trabecular bone sites; however, catabolism was more pronounced in the hypercalcemic group.^10^ The normocalcemic group had greater preservation of cancellous bone, indicating differences in the extent and site of catabolism depending on the form of primary hyperparathyroidism.^10^

Previous reports have described the “salt and pepper” appearance of the calvarium in cases of hypercalcemic primary hyperparathyroidism and secondary hyperparathyroidism but typically not in cases of normocalcemia.^11^, ^12^, ^13^ However, 1 case report described a case of mandibular brown tumor (osteitis fibrosa cystica) in a patient who was ultimately found to have normocalcemic hyperparathyroidism but notably in the setting of 25-hydroxyvitamin D deficiency and parathyroid adenoma.^14^ Although the patient in the abovementioned case report shares similarities with the patient presented here, the crucial difference is that secondary causes of hyperparathyroidism were excluded in the case of our patient.

Considering our patient’s “salt and pepper” lesions, it is important to recall that the calvarium is composed of 2 layers of cortical bone, separated by trabecular (cancellous) bone.^15^ The “salt and pepper” appearance represents multiple small hyperlucencies caused by the resorption of trabecular bone due to increased osteoclast activity.^16^ It was surprising that the patient did not have other signs of skeletal involvement, such as fractures. However, the patient had reduced bone mineral density at the femoral neck, which contains substantial cortical bone, which tends to be affected in normocalcemic hyperparathyroidism. It is unclear why the patient had significant trabecular bone loss, as it contradicts the general trend seen in the literature regarding cortical bone loss, particularly if the patient had a long, early subclinical phase (involving bone loss) that is a part of the new conceptualization involved in the changing chronology of normocalcemic primary hyperparathyroidism.^17^, ^18^, ^19^

## Conclusion

This report highlights a rare presentation of normocalcemic primary hyperparathyroidism diagnosed after imaging revealed “salt and pepper” lesions of the calvarium. These lesions have traditionally been demonstrated in patients with hypercalcemic primary hyperparathyroidism or secondary hyperparathyroidism. We are unaware of a similar case of normocalcemic primary hyperparathyroidism in which the presenting clinical phenotype involved radiographic findings of bone lesions alone. Understanding atypical presentations of the disease will be helpful as we explore our changing understanding of the various clinical features and trajectory of normocalcemic primary hyperparathyroidism.

## Disclosure

The authors have no multiplicity of interest to disclose.
